# A Universal Spinning‐Coordinating Strategy to Construct Continuous Metal–Nitrogen–Carbon Heterointerface with Boosted Lithium Polysulfides Immobilization for 3D‐Printed Li—S Batteries

**DOI:** 10.1002/advs.202203181

**Published:** 2022-07-21

**Authors:** Yue Ouyang, Wei Zong, Xiaobo Zhu, Lulu Mo, Guojie Chao, Wei Fan, Feili Lai, Yue‐E Miao, Tianxi Liu, Yan Yu

**Affiliations:** ^1^ State Key Laboratory for Modification of Chemical Fibers and Polymer Materials College of Materials Science and Engineering Donghua University 2999 North Renmin Road Shanghai 201620 P. R. China; ^2^ The Key Laboratory of Synthetic and Biological Colloids Ministry of Education School of Chemical and Material Engineering Jiangnan University Wuxi, Jiangsu 214122 P. R. China; ^3^ Department of Chemistry KU Leuven Celestijnenlaan 200F Leuven 3001 Belgium; ^4^ Hefei National Research Center for Physical Sciences at the Microscale Department of Materials Science and Engineering National Synchrotron Radiation Laboratory CAS Key Laboratory of Materials for Energy Conversion University of Science and Technology of China Hefei Anhui 230026 P. R. China

**Keywords:** 3D printing technique, electrospun porous fibers, lithium‐sulfur batteries, metal–nitrogen–carbon heterointerface, strongly coupled structure

## Abstract

Constructing intimate coupling between transition metal and carbon nanomaterials is an effective means to achieve strong immobilization of lithium polysulfides (LiPSs) in the applications of lithium–sulfur (Li—S) batteries. Herein, a universal spinning‐coordinating strategy of constructing continuous metal–nitrogen–carbon (M—N—C, M = Co, Fe, Ni) heterointerface is reported to covalently bond metal nanoparticles with nitrogen‐doped porous carbon fibers (denoted as M/M—N@NPCF). Guided by theoretical simulations, the Co/Co—N@NPCF hybrid is synthesized as a proof of concept and used as an efficient sulfur host material. The polarized Co—N—C bridging bonds can induce rapid electron transfer from Co nanoparticles to the NPCF skeleton, promoting the chemical anchoring of LiPSs to improve sulfur utilization. Hence, the as‐assembled Li—S battery presents a remarkable capacity of 781 mAh g^−1^ at 2.0 C and a prominent cycling lifespan with a low decay rate of only 0.032% per cycle. Additionally, a well‐designed Co/Co—N@NPCF‐S electrode with a high sulfur loading of 7.1 mg cm^−2^ is further achieved by 3D printing technique, which demonstrates an excellent areal capacity of 6.4 mAh cm^−2^ at 0.2 C under a lean‐electrolyte condition. The acquired insights into strongly coupled continuous heterointerface in this work pave the way for rational designs of host materials in Li—S systems.

## Graphical abstract

A strongly coupled hybrid with continuous metal–nitrogen–carbon—— heterointerface (M/M─N@NPCF, M = Co, Fe, Ni) is developed by a universal spinning‐coordinating strategy, which can achieve efficient charge transfer and boost lithium polysulfides immobilization. Combined with 3D printing technique, the Co/Co—N@NPCF hybrid as a proof of concept shows excellent electrochemical performance even under high sulfur loadings and lean‐electrolyte conditions.

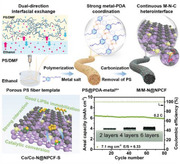

## 1 Introduction

Lithium–sulfur (Li—S) batteries have received great attention among the secondary batteries owing to their inherent high specific capacity of 1675 mAh g^−1^ and energy density of 2600 Wh kg^−1^.^[^ ^1^, ^2^, ^3^, ^4^, ^5^ ^]^ Besides, other appealing features such as natural abundance, low cost, and nontoxicity of sulfur further bring extra benefits to the market potential of Li—S batteries.^[^ ^6^, ^7^, ^8^ ^]^ However, their practical applications are still hindered by three main problems, including the poor conductivity of sulfur and lithium sulfide (Li_2_S), the severe shuttling effect derived from the dissolution of intermediate lithium polysulfides (LiPSs), and the huge volumetric expansion (≈80%) of sulfur cathode upon cycling processes.^[^ ^9^, ^10^, ^11^ ^]^ Consequently, such drawbacks cause sluggish redox reaction kinetics, irreversible loss of active material, finally leading to fast capacity fading, and poor cyclic lifespan.^[^ ^12^, ^13^, ^14^ ^]^

To address the above issues, advanced sulfur host materials, like highly conductive matrixes with adsorption/catalytic conversion capacities for LiPSs, have been constructed.^[^ ^15^ ^]^ In particular, carbonaceous materials, possessing good mechanical stability, excellent conductivity, and reproducible properties, were beneficial to serving as host materials for sulfur species. Nonetheless, the weak van der Waals interactions between nonpolar carbon and polar LiPSs could only mitigate the undesirable LiPSs shuttling to a certain extent.^[^ ^16^, ^17^, ^18^ ^]^ And the poor conversion kinetics from long‐chain LiPSs to the final Li_2_S still limit the high power output of the batteries, especially those with high sulfur loadings or low electrolyte/sulfur ratios.^[^ ^19^ ^]^ In light of this, combining highly active transition metal catalysts (e.g., Fe, Co, and Ni) with carbonaceous materials have been considered a promising strategy to effectively encapsulate LiPSs, and reduce the activation energy of the electrocatalytic reactions.^[^ ^20^, ^21^, ^22^, ^23^ ^]^ One can expect that in the metal/carbonaceous heterostructures, enhanced LiPSs immobilization and boosted redox kinetics could be achieved to maximize the sulfur utilization of Li—S batteries.^[^ ^24^, ^25^ ^]^ Moreover, combining transition metals with carbonaceous materials is an effective approach to improve its structural stability.^[^ ^26^, ^27^ ^]^ Nevertheless, it is still difficult to achieve the effective charge transfer and tunable electronic properties between metallic nanoparticles and carbonaceous materials due to very poor contact. It was of great meaning to construct effective bridging bonds at the heterointerface, which could improve the interfacial connections between the two different phases. In particular, the accelerated reaction kinetics with abundant electron transfer paths at an atomic level can benefit for enhancing energy storage properties. However, the rational construction of robust coupling interactions between transitional metals and the carbon matrix still remains a considerable challenge to realize the tunable interfacial and electronic properties for boosted transfer kinetics.

Another concern related to Li—S batteries lies in the difficulty of constructing high sulfur loading cathodes to simultaneously achieve high areal capacities together with excellent cycling stability, as the thick electrodes fabricated via the traditional blade‐casting route are prone to cracking and peeling off.^[^ ^28^, ^29^ ^]^ The slurry coating‐derived plain electrodes are also not beneficial to electrolyte infiltration and electron/ion transport, resulting in poor electrochemical performance.^[^ ^30^ ^]^ Recently, the 3D printing technique offers a feasible solution to scalable manufacturing customizable energy storage systems for environmental friendliness and cost‐effectiveness.^[^ ^31^, ^32^, ^33^ ^]^ It allows precise regulation of geometry and structure in preparing electrodes with controllable active material loading and enhanced electron/ion conductivity, thereby effectively improving the capacity output of Li—S batteries.^[^ ^34^, ^35^, ^36^ ^]^ Nevertheless, the rational design and employment of 3D‐printed scaffolds to construct high‐performance Li—S batteries are still in their infancy.

Herein, we report a general strategy of constructing continuous metal–nitrogen–carbon (M—N—C, M = Co, Fe, Ni) heterointerface, by taking electrospun 1D porous fibers as the template and in situ polymerized polydopamine (PDA) as the interfacial coordination linker, to covalently bond electrocatalytic metal nanoparticles onto nitrogen‐doped porous carbon fibers (denoted as M/M—N@NPCF) through a simple carbonization treatment. Benefitting from the excellent adhesion and strong coordination of PDA towards fibrous template and metal ions, the synthetic method is highly universal for incorporating various metal nanoparticles onto the 1D carbon fibers to form continuous metal–nitrogen–carbon heterointerfaces. The effective M—N—C bridging bonds then can manipulate electron redistribution at the heterointerface, and induce efficient electron transfer from metal nanoparticles to the NPCF skeleton for tightly anchoring LiPSs by Lewis acid–base interactions. Meanwhile, the abundant metal nanoparticles embedded within the NPCF skeleton can function as the electrocatalyst to trigger rapid LiPSs conversion and further enhance sulfur utilization. As a proof of concept, the Co/Co—N@NPCF hybrid as sulfur host exhibits an excellent rate capability of 781 mAh g^−1^ at 2.0 C and a stable cycling lifespan with 90.4% retention after 300 cycles. More impressively, the printable Co/Co—N@NPCF‐S electrodes possessing elevated sulfur loadings and enhanced electron/ion transport are enabled by a 3D printing technique, delivering an excellent areal capacity of 6.4 mAh cm^−2^ at 0.2 C with a high sulfur loading of 7.1 mg cm^−2^.

## 2 Results and Discussion

**Figure 1:**
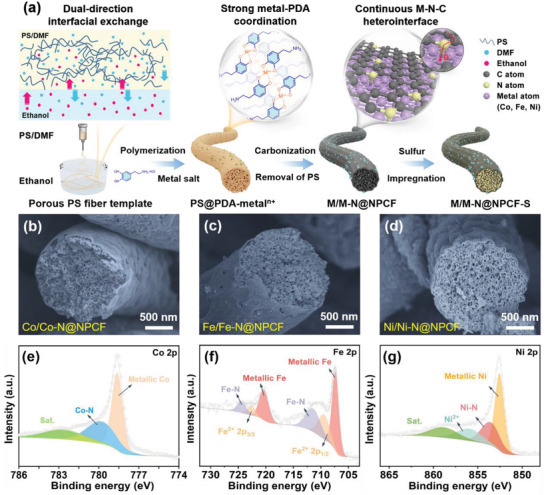
a) Schematic illustration of the preparation of the M/M—N@NPCF‐S composite. b–d) SEM images and e–g) XPS spectra of M/M—N@NPCF hybrids: b,e) Co/Co—N@NPCF, c,f) Fe/Fe‐N@NPCF, and d,g) Ni/Ni‐N@NPCF.

The general synthetic process for the M/M—N@NPCF‐S composite is schematically illustrated in **Figure** **1**a. Different from the traditional electrospinning method, an ethanol solution is used as the nonsolvent receiving bath to collect polystyrene (PS) fibers. When the fluid PS jet touches the ethanol solution, a dual‐direction solvent exchange occurs between *N,N*‐dimethylformamide and ethanol molecules. Afterward, the concentrated polymer‐rich domain in the phase‐separated spinning jet is solidified into the fiber skeleton while the original solvent‐rich domain transforms into the pores, and eventually forms mesoporous PS fibers as shown in Figure S1a,b, Supporting Information. The as‐obtained PS fibers were then put into an aqueous solution containing dopamine and metal salt. Owing to the high affinity of dopamine towards both the PS fiber template and metal ions, in situ self‐polymerization of dopamine and metal ion coordination takes place on the PS fibers to obtain PS@PDA‐metal*^n^* ^+^ fibers, which exhibit smooth surface and integrally maintain the original porous microstructure of PS fibers (Figure S1c,d, Supporting Information). After the high‐temperature carbonization in N_2_ atmosphere, porous PS fibers are basically decomposed as the sacrificial template, while the reduced metal nanoparticles form a strongly coupled M—N—C bridging heterointerface with the NPCF skeleton derived from the PDA coating layer, which finally produces the M/M—N@NPCF hybrid. As shown in the scanning electron microscopy (SEM) images of Figure 1b–d and Figure S2, Supporting Information, the nanofibrous morphology and intact porous structure of M/M—N@NPCF hybrids (M = Co, Fe, Ni) are well retained under the protection of the thin PDA‐derived carbon wall after removing the PS template. Compared with the uniform distribution of C, Co, and N elements within the fibrous framework of Co/Co—N@NPCF hybrid (Figure S3, Supporting Information), the nitrogen doped carbon embedded with cobalt nanoparticles (Co/Co—N@NC) prepared without the addition of PS fiber template appears as agglomerates composed of carbon spheres with different sizes (Figure S4, Supporting Information). And the bonding configuration and chemical composition of M/M—N@NPCF hybrids were analyzed by using X‐ray photoelectron spectroscopy (XPS). The full survey spectrum in Figure S5, Supporting Information, reveals the co‐existence of C, N, and metal (Co, Fe, Ni) species in M/M—N@NPCF hybrids. The curve‐fitted Co 2p^3/2^ spectrum in Figure 1e exhibits two types of Co species, including the metallic Co at 778.6 eV, which proves the existence of cobalt nanoparticles. Another peak at 780.1 eV can be ascribed to the Co—N bond deriving from Co^2+^‐catechol bis‐complex, which contributes to the formation of the continuous Co—N—C heterointerface between Co nanoparticles and the NPCF matrix. The Raman spectroscopy was also performed to further detect Co—N—C heterointerface. As shown in Figure S6, Supporting Information, two peaks at 1331 and 1591 cm^−1^ can be clearly observed in NPCF, which correspond to the D and G bands from graphitic carbon, respectively. Typically, the D band is considered the defect mode while the G band represents stretching mode in the graphite plane. The obvious blue‐shifting of the G band peak in Co/Co—N@NPCF hybrid indicates the strong interaction and rapid electron transport between Co nanoparticles and NPCF matrix. In addition, Fe/Fe‐N@NPCF and Ni/Ni‐N@NPCF hybrids are also confirmed to possess strongly coupled metal–nitrogen–carbon heterointerface (Figure 1f,g), which can facilitate the charge transfer between metal and NPCF, so as to accelerate reaction kinetics in the further electrochemical process. The above results indicate that the continuous M—N—C heterointerface based on 1D electrospun fibrous templates can be successfully achieved for multiple metals.

**Figure 2:**
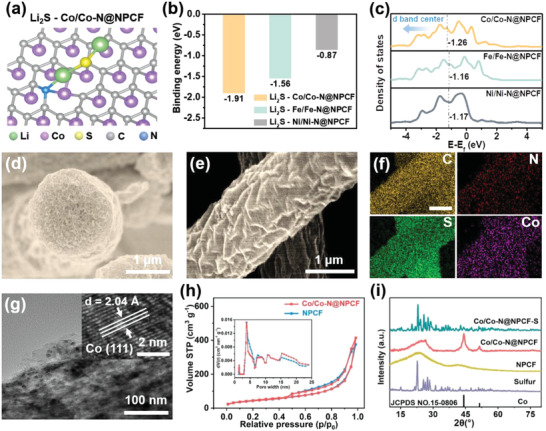
a) Adsorption configuration of Li_2_S —Co/Co—N@NPCF. b) Absorption energy with Li_2_S, and c) DOS analyses of Co/Co—N@NPCF, Fe/Fe‐N@NPCF, and Ni/Ni‐N@NPCF, respectively. d–e) SEM images and f) corresponding EDX elemental mappings of Co/Co—N@NPCF‐S composite. g) TEM and HRTEM (inset) images of Co/Co—N@NPCF‐S composite. h) Nitrogen adsorption‐desorption isotherms and pore size distributions (inset) of Co/Co—N@NPCF and NPCF. i) XRD patterns of sulfur, NPCF, Co/Co—N@NPCF, and Co/Co—N@NPCF‐S composites.

To select the most promising transition metal species in various M/M—N@NPCF hybrids, density functional theory (DFT) calculations were carried out. As displayed in **Figure** **2**a and Figure S7, Supporting Information, the optimized adsorption configurations of the discharge product of Li_2_S on M/M—N@NPCF hybrids (M = Co, Fe, Ni) are constructed. Figure 2b shows that the Co/Co—N@NPCF hybrid has the strongest binding energy with Li_2_S (−1.91 eV). In quantum chemistry, the formation and breakage of chemical bonds between two elements are closely related to the valence electrons of the element (*d*‐band electrons for metal elements, *p*‐band electrons for nonmetal elements).^[^ ^37^ ^]^ To uncover the adsorption essence, the density of state analyses and calculated *d*‐band center were carried out for these hybrids (Figure 2c). It can be observed that the *d*‐band center in Co/Co—N@NPCF has a distinct downshift with respect to the Fermi level, which represents a reduced filling of the anti‐bonding states, and thus promoting the binding between the Co/Co—N@NPCF hybrid and the adsorbate. Herein, the Co/Co—N@NPCF hybrid with the greatest potential was chosen as the sulfur host to prepare the Co/Co—N@NPCF‐S composite cathode via a molten diffusion method for subsequent analyses. No obvious sulfur bulk is observed in the Co/Co—N@NPCF‐S composite according to the SEM images (Figure 2d,e) and corresponding EDX mappings (Figure 2f; Figure S8, Supporting Information), demonstrating the successful diffusion and encapsulation of sulfur in the host material. Moreover, the transmission electron microscopy (TEM) images in Figure 2g clearly exhibit that crystalline Co nanoparticles with an average diameter of 10 nm are uniformly distributed in the NPCF matrix without agglomeration, which is attributed to the coordinated interactions between cobalt ions and catechol groups. The NPCF‐S composite was achieved through the same process for comparison (Figure S9, Supporting Information).

Given that high porosity of the host is in favor of immobilizing LiPSs and improving cycling stability, the specific surface area was analyzed by N_2_ adsorption‐desorption isotherms. As shown in Figure 2h, the Co/Co—N@NPCF hybrid exhibits a high Brunauer–Emmett–Teller (BET) surface area of 117.8 m^2^ g^−1^ with abundant micro‐ and mesopores, which is attributed to the solvent exchange process at the spinning jet‐ethanol interface. X‐ray diffraction (XRD) technique is also valid to characterize the structures of diverse samples. Obviously, two apparent diffraction peaks at 2*θ* = 44.2° and 51.6° in the XRD pattern of the Co/Co—N@NPCF hybrid (Figure 2i) are ascribed to the (111) and (200) lattice facets of cobalt phase (JCPDS no. 15‐0806) respectively, indicating the successful reduction of Co ions to form Co nanoparticles in the frameworks. Strong diffraction peaks corresponding to sulfur are also detected in the Co/Co—N@NPCF‐S composite, demonstrating that sulfur is successfully encapsulated within the host material. The corresponding loading amount of sulfur in Co/Co—N@NPCF‐S and NPCF‐S composites are quantitatively analyzed to be 65 wt% and 63 wt% by thermogravimetric analyses (TGA) in Figure S10, Supporting Information, respectively.

**Figure 3:**
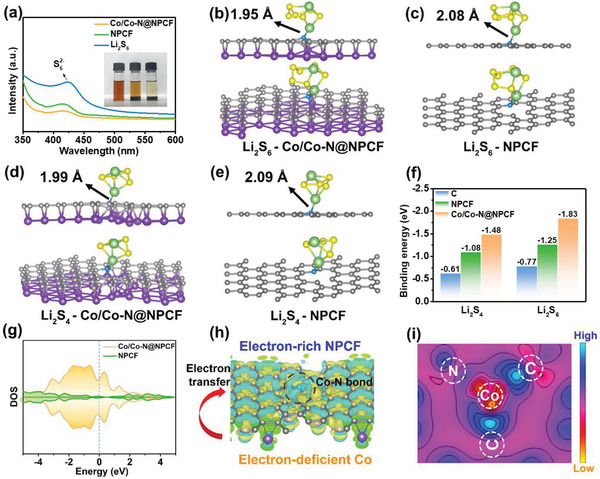
a) The digital photographs and UV–vis spectra of different samples after static adsorption of Li_2_S_6_ (from left to right: blank, NPCF, Co/Co—N@NPCF samples). Adsorption configurations of b) Li_2_S_6_ —Co/Co—N@NPCF, c) Li_2_S_6_ —NPCF, d) Li_2_S_4_ —Co/Co—N@NPCF, and e) Li_2_S_4_ —NPCF. The Li, C, N, S, and Co atoms are denoted by green, grey, blue, yellow, and purple balls, respectively. f) Adsorption energy of different configurations. g) Density of states projected to the *p_z_* orbital of N atoms in NPCF and Co/Co—N@NPCF, respectively. h) Differential charge density and i) 2D charge map of Co/Co—N@NPCF hybrid.

To intuitively investigate the anchoring capability of the Co/Co—N@NPCF hybrid towards LiPSs, visualized adsorption tests are carried out in parallel by adding identical amounts of the samples in 5 mM Li_2_S_6_ solution. It is noticeable that the Co/Co—N@NPCF group becomes nearly transparent after immersing for 12 h, while the NPCF group remains slightly yellow in the inset of **Figure** **3**a. The corresponding residual concentration of Li_2_S_6_ is analyzed by ex situ ultraviolet–visible (UV–vis) absorption spectroscopy. Consistent with the degree of color fading, the discernable peak intensity of S_6_^2−^ at approximately 415 nm declines drastically with the addition of the Co/Co—N@NPCF hybrid. This implies a much stronger absorptivity of Co/Co—N@NPCF towards LiPSs than the ordinary NPCF nanofibers, which would be beneficial for the effective suppression of shuttle effect during the charge/discharge process. To further explore the anchoring mechanism of the Co/Co—N@NPCF hybrid for LiPSs, DFT calculations are implemented to model the adsorption of Li_2_S*_n_* (*n* = 4 and 6) on the surfaces of Co/Co—N@NPCF, NPCF, and C configurations (Figure 3b–e; Figure S11, Supporting Information). Comparatively, the adsorption capability of Li_2_S*_n_* on Co/Co—N@NPCF is stronger than that on NPCF as shown in Figure 3f. In particular, the adsorption energy of Co/Co—N@NPCF toward Li_2_S_6_ is calculated to be −1.83 eV, which is superior to that of NPCF (−1.25 eV) or carbon (−0.77 eV). According to the charge density difference (Figure 3h), electrons are transferred from the neighboring Co atom to NPCF matrix via the electron bridge of Co—N—C, thus facilitating the efficient electron transfer at the heterogeneous interface.^[^ ^26^, ^38^ ^]^ The color in the corresponding 2D charge map (Figure 3i) further suggests the degree of charge accumulation in this region. It is obvious that the transferred electrons from the electron‐deficient Co atom (yellow region) enable the N atom (blue region) to possess extra‐pair electrons. The highly electron‐rich N heteroatom could form Li—N bonds with the Li atom in Li_2_S_6_ through Lewis acid–base interactions, and thus realizing the strong immobilization of LiPSs on the Co/Co—N@NPCF hybrid. As a result, the Co/Co—N@NPCF‐S electrode presents a lower shuttle current of 5.5 × 10^−4^ mA cm^−2^ than NPCF‐S (2.2 × 10^−3^ mA cm^−2^) after current stabilization (Figure S12, Supporting Information), confirming the strong ability of Co/Co—N@NPCF hybrid in high‐efficiency suppression of LiPSs shuttling in the Li—S system.

**Figure 4:**
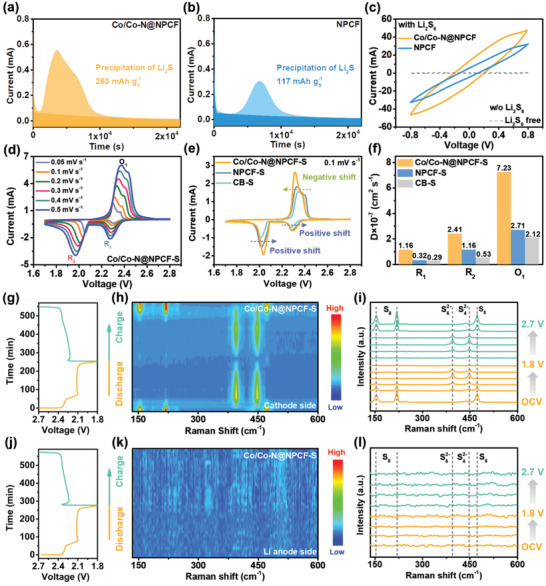
a,b) Potentiostatic discharging profiles of Li_2_S_8_ solution on Co/Co—N@NPCF and NPCF surfaces at 2.05 V. c) CV profiles of Co/Co—N@NPCF and NPCF‐based symmetric cells. d) CV curves of Co/Co—N@NPCF‐S electrode under different scanning rates. e) CV curves at 0.1 mV s^−1^, and f) Li‐ion diffusion coefficient of Co/Co—N@NPCF‐S, NPCF‐S, and CB‐S electrodes. The voltage–time curves and corresponding in situ Raman spectra collected at (g–i) the surface of the Co/Co—N@NPCF‐S cathode, and (j–l) the separator facing the Li anode side, respectively.

Note that the entrapment of LiPSs on the surface of Co/Co—N@NPCF hybrid constitutes the prerequisite for their rapid conversion. To get full insight into the electrocatalytic behaviors of the Co/Co—N@NPCF hybrid on sulfur redox kinetics, a series of electroanalytic measurements is further conducted. According to the theory that 75% of the discharge capacity generates from the conversion process of Li_2_S_4_ to Li_2_S,^[^ ^39^, ^40^ ^]^ the amount of Li_2_S precipitation on the substrate was measured to evaluate the catalytic effect of the Co/Co—N@NPCF hybrid. Typically, coin cells were assembled using Co/Co—N@NPCF or NPCF as the cathode, bare Li foil as the anode, and a Li_2_S_8_/tetraglyme solution as the electrolyte. As shown in **Figure** **4**a,b, the Co/Co—N@NPCF electrode reaches a higher Li_2_S nucleation capacity of 263 mAh g_S_^−1^ than the NPCF electrode of 117 mAh g_S_^−1^, which elucidates that the redox reaction of LiPSs is strongly triggered by Co nanoparticles within the NPCF matrix. The cyclic voltammetry (CV) of the symmetric cells was also adopted to analyze the promoted catalytic performance of Co/Co—N@NPCF in Li—S chemistry, in which Co/Co—N@NPCF was employed both as the working and counter electrodes with Li_2_S_6_ electrolyte. The Co/Co—N@NPCF hybrid leads to a much higher CV current response compared to NPCF under identical conditions in the CV profiles (Figure 4c). Additionally, the Co/Co—N@NPCF hybrid exhibits an upper response current and smaller Tafel slope than those of NPCF under cathodic and anodic scans in Tafel plots (Figure S13, Supporting Information). All of these results manifest the enhanced electrocatalytic activity of Co/Co—N@NPCF towards sulfur species.

Furthermore, CV curves of the fabricated cathode materials in a range of 1.7–2.8 V were collected under various scan rates (0.05–0.5 mV s^−1^) to manifest the specific electrochemical reaction process (Figure 4d; Figure S14, Supporting Information). Two cathodic peaks (R_1_ and R_2_) can be ascribed to the reduction of S_8_ to long‐chain Li_2_S*_n_* (4 ≤ *n* ≤ 8) and the further reduction to solid‐state Li_2_S_2_/Li_2_S. The anodic peak (O_1_) is attributed to the oxidation of Li_2_S/Li_2_S_2_ to LiPSs and eventually to sulfur. Taking the CV profiles at the scan rate of 0.1 mV s^−1^ as an example, the Co/Co—N@NPCF‐S electrode shows an obvious positive shift of cathodic peaks and negative shift of anodic peak compared to NPCF‐S and CB‐S, accompanied with an increased peak intensity (Figure 4e), which indicates a lower polarization. Besides, the Tafel slopes are calculated to quantify the catalytic activities. As shown in Figure S15, Supporting Information, Tafel slopes of the reduction and oxidation peaks are only 17.8 and 46.5 mV dec^−1^ for the Co/Co—N@NPCF‐S electrode respectively, which stay much lower than those of NPCF‐S (24.3 and 66.5 mV dec^−1^) and CB‐S electrodes (27.6 and 76.3 mV dec^−1^), implying the conspicuous electrocatalytic efficiency in both reduction and oxidation processes with the presence of Co/Co—N@NPCF hybrid. Such substantially accelerated conversion kinetics can be observed in the in‐depth charge/discharge curves (Figure S16, Supporting Information). Attributing to the reduced electrochemical polarization and promoted redox kinetics stemming from the robust electrocatalytic effect of Co nanoparticles, the Co/Co—N@NPCF‐S electrode delivers the smallest voltage gap (0.13 V) among various cathode materials. Moreover, the Li_2_S nucleation kinetics can be evaluated based on the voltage difference between the Li_2_S nucleation point and the second voltage platform. The Co/Co—N@NPCF‐S electrode renders a lower overpotential of only 10 mV, compared with the NPCF‐S electrode (25 mV), indicating the faster kinetics and smaller energy barrier of Li_2_S nucleation for Co/Co—N@NPCF hybrid, which is in accordance with the Li_2_S precipitation experiments in Figure 4a,b. The lower activation barrier of Li_2_S can also be seen for the Co/Co—N@NPCF‐S electrode during the charge process. Meanwhile, the Li‐ion diffusion coefficients (*D*_Li_) can be derived from CV curves under different sweep rates based on the Randles–Sevcik equation:^[^ ^41^, ^42^ ^]^

(1) $$I_{p}=\left(2.69\times 10^{5}\right)n^{1.5}AD^{0.5}v^{0.5}C$$

in which *I*_p_ is the current of peaks, *v* is the scan rate, *n* is the number of reaction charges, *A* stands for the area of electrode, and *C* is the concentration of Li^+^ in the electrolyte. The linear fitting of *I*_p_ versus *v* ^1/2^ for various electrodes is plotted in Figure S17, Supporting Information, and the Co/Co—N@NPCF‐S electrode always shows higher curve slopes than those of NPCF‐S and CB‐S electrodes, suggesting faster ion diffusion and LiPSs transformation (Figure 4f). In addition, the Co/Co—N@NPCF‐S electrode has a lower interfacial charge transfer resistance (*R*_ct_) than those of NPCF‐S and CB‐S electrodes according to the electrochemical impedance spectroscopy (EIS) of Li—S batteries (Figure S18 and Table S1, Supporting Information). This suggests the largely accelerated charge transfer at the electrode‐polysulfides interface, which can considerably facilitate the liquid–solid conversion and effectively inhibit LiPSs migration.

In situ Raman spectroscopy was further performed to trace the charge/discharge processes in Li—S batteries with Co/Co—N@NPCF‐S as the cathode (Figure S19, Supporting Information). It is worth noting that the Raman signal was collected from the surface of the cathode (Figure 4g–i) and the separator facing the Li anode side (Figure 4j–l), respectively, to real‐time monitor the transformation of sulfur species and shuttle status of LiPSs.^[^ ^43^ ^]^ At the beginning of the discharge process (Figure 4h,i), the characteristic peaks of S_8_ located at 154.2, 219.3, and 472.5 cm^−1^ gradually disappear, while the characteristic peaks of LiPSs occurred at 394.8 and 448.4 cm^−1^ constantly strengthen. As the discharge proceeds, the signals of LiPSs fade away due to the complete conversion to Li_2_S. During the subsequent charge process, the Raman peaks of LiPSs reappear and become stronger until the signals of S_8_ finally arise, which corresponds exactly to the CV and discharge/charge curves. Additionally, no obvious signal of LiPSs is detected on the surface of the separator which faces the Li metal anode side throughout the entire discharge/charge process (Figure 4k,l). The above records of Raman signal changes indicate that Co/Co—N@NPCF hybrid can regulate the transformation of sulfur species and restrict the shuttling effect of LiPSs upon cycling, which is expected to achieve a high sulfur utilization in Li—S batteries.

**Figure 5:**
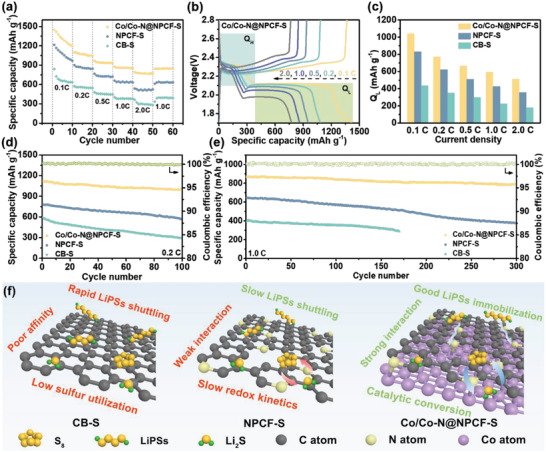
a) Rate capabilities, b) galvanostatic charge/discharge curves, and c) comparison of the *Q*_L_ capacities of Co/Co—N@NPCF‐S, NPCF‐S, and CB‐S electrodes. d) Cycling performance at 0.2 C and e) long‐term cycling stability at 1.0 C of Co/Co—N@NPCF‐S, NPCF‐S, and CB‐S electrodes. f) Schematic of LiPSs immobilization for Co/Co—N@NPCF‐S composites.

Based on the great encapsulation of the Co/Co—N@NPCF hybrid for LiPSs, the electrochemical performances were systematically studied with Li foil acting as the anode in Li—S batteries. As presented in **Figure** **5**a, the rate capabilities of different samples were measured under diverse current densities from 0.1 to 2.0 C. It can be observed that the Co/Co—N@NPCF‐S electrode furnishes high reversible capacities of 1476, 1098, 947, 861, and 781 mAh g^−1^ when cycling at 0.1, 0.2, 0.5, 1.0, and 2.0 C, respectively. As the current density switches back to 1.0 C, a high capacity of 845 mAh g^−1^ can still be recovered, corresponding to 98.1% retention of previous capacity at the same C‐rate. This superior rate performance is much higher than those of NPCF‐S and CB‐S, which suggests the high electrical conductivity and good structural stability with splendid electrocatalytic activity of the Co/Co—N@NPCF hybrid. Likewise, Fe/Fe—N@NPCF—S and Ni/Ni—N@NPCF—S electrodes also present decent rate performance (Figure S20, Supporting Information). Moreover, the improvement in the electrochemical performance supported by the enhanced redox kinetics is well reflected in the galvanostatic charge‐discharge (GCD) curves of Co/Co—N@NPCF—S electrode at different current densities (Figure 5b). Obviously, all the discharge curves of the Co/Co—N@NPCF—S electrode exhibit two distinct plateaus including high discharge plateau (*Q*_H_) and low discharge plateau (*Q*_L_) even at high current rates. Clearly, the Co/Co—N@NPCF—S electrode delivers the highest *Q*_H_ and *Q*_L_ capacities among the three electrodes (Figure 5c and Figure S21, Supporting Information), confirming the superior activity in catalyzing the reduction of S_8_ to LiPSs and further to Li_2_S.

The suppressed shuttle effect and boosted redox dynamics also endow the Co/Co—N@NPCF‐S electrode with excellent cyclic stability. As shown in Figure 5d, steady cycling of the Co/Co—N@NPCF‐S electrode can be observed at 0.2 C, reaping a high initial capacity of 1115 mAh g^−1^ with 90.4% retention after 100 cycles. This cycling performance markedly exceeds the initial capacities of 778 and 582 mAh g^−1^ for NPCF‐S and CB‐S electrodes with only 72.3% and 50.7% retentions after 100 cycles, respectively. When the current density is enlarged to 1.0 C (Figure 5e), the Co/Co—N@NPCF‐S electrode affords a high initial capacity of 867 mAh g^−1^ with a Coulombic efficiency (CE) of nearly 100% and an inappreciable capacity decay rate of 0.032% per cycle for 300 cycles, while the NPCF‐S electrode presents an inferior cycling stability (decay rate of 0.138% per cycle). Meanwhile, Co/Co—N@NPCF electrode can still achieve decent retention of 86.5% after 500 cycles at 2.0 C (Figure S22, Supporting Information). Such high capacity retentions for the Co/Co—N@NPCF‐S electrode at different C‐rates, clearly demonstrate the successful trapping of LiPSs via physical adsorption by the porous framework and Lewis acid–base interactions of the Co—N—C heterointerface. Furthermore, the surface morphologies of Co/Co—N@NPCF‐S electrode and the corresponding Celgard separator after 100 cycles at 1.0 C were also investigated. As shown in Figure S23a,b, Supporting Information, the Co/Co—N@NPCF‐S composite remains a relatively intact nanofibrous structure with uniformly distributed sulfur, while the separator facing the cathode side still remains a typical porous structure without any visible solid particles after cycling. On the contrary, the conventional CB‐S electrode possesses severe macroscopic cracks and the pores of Celgard separator are almost blocked by a mass of Li_2_S_2_/Li_2_S nanoparticles (Figure S23c,d, Supporting Information), denoting a vast loss of sulfur along with a negative influence on Li^+^ transport and thus resulting in the low specific capacity and inferior cycle stability. Compared to CB with poor structural stability and NPCF matrix with fewer adsorptive/catalytic sites, the continuous Co—N—C heterointerface and Co nanoparticles in the strongly coupled Co/Co—N@NPCF hybrid can enhance LiPSs anchoring and expedite redox kinetics to efficiently mitigate the shuttling effect. The mesoporous carbon nanofibers prepared based on the novel electrospinning strategy can also effectively confine sulfur species via spatial restriction and relieve volume variation during the cycling process. As a result, Li—S batteries with high utilization of active material, fabulous rate performance, and prominent cycling lifespan can be achieved (Figure 5f).

**Figure 6:**
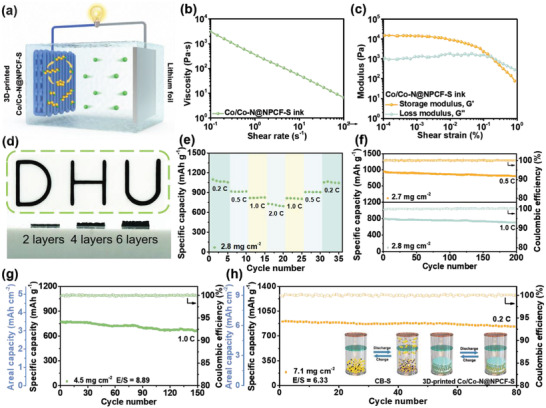
a) Schematic illustration of 3D‐printed Co/Co—N@NPCF‐S electrode applied in Li—S batteries with optimized LiPSs immobilization and conversion. b) Apparent viscosity, c) storage modulus (G′), and loss modulus (G″) of the Co/Co—N@NPCF‐S ink. d) Digital photos of 3D‐printed Co/Co—N@NPCF‐S architectures. e) Rate and f) cycling performance of 3D‐printed Co/Co—N@NPCF‐S electrodes with the sulfur loading of around 2.8 mg cm^−2^. Cycling performance of 3D‐printed Co/Co—N@NPCF‐S electrodes with sulfur loadings of g) 4.5 mg cm^−2^ and h) 7.1 mg cm^−2^, respectively.

To translate the superiority of the strongly coupled Co/Co—N@NPCF hybrid with continuous Co—N—C heterointerface into practical applications, the 3D printing technique is employed to fabricate sulfur cathodes with controllable sulfur loadings and customizable structures in **Figure** **6**a. The rheological properties of the as‐prepared ink consisting of Co/Co—N@NPCF‐S and GO suspensions are examined in advance to ensure a favorable 3D printing process. As depicted in Figure 6b, the typical shear‐thinning non‐Newtonian behavior renders the continuous flow of the Co/Co—N@NPCF‐S based ink upon extrusion. The ink exhibits a relatively high viscosity over 10^2^ Pa·s at a shear rate of 1 s^−1^, which can improve its printability to prepare complex patterns and structures. Besides, the variation trends of the storage modulus (G′) and loss modulus (G″) versus shear stress shown in Figure 6c enable a stable architecture after extrusion without collapse.^[^ ^44^ ^]^ Based on the above features, the 3D‐printed architectures with various patterns such as “D, H, U” or multiple layers can be easily realized (Figure 6d), presenting a high printing versatility. The top‐view SEM image in Figure S24, Supporting Information, further reveals a stack structure for the freeze‐dried free‐standing electrode.

The 3D‐printed Co/Co—N@NPCF‐S electrodes possessing sulfur loadings of 2.8–7.1 mg cm^−2^ can be directly constructed by varying the printing layer numbers. As shown in Figure 6e, the 3D‐printed Co/Co—N@NPCF‐S electrode with a sulfur loading of 2.8 mg cm^−2^ delivers high capacities of 1069, 914, 821, and 723 mAh g^−1^ at 0.2, 0.5, 1.0 and 2.0 C, respectively. When turning back to 0.2 C, a reversible capacity of 1055 mAh g^−1^ can still be harvested. The two discharging plateaus can be clearly observed in Figure S25, Supporting Information, even at a high current rate of 2.0 C, indicating the remarkable rate capability owing to the accelerated redox kinetics. The 3D‐printed Co/Co—N@NPCF‐S electrode with around 2.8 mg cm^−2^ also presents outstanding cycling performance with capacity retentions of 88.5% and 85.3% at both 0.5 and 1.0 C, respectively (Figure 6f), which is superior to the traditional plain Co/Co—N@NPCF‐S electrodes (Figure S26, Supporting Information). When the sulfur loading further increases to 4.5 mg cm^−2^, the 3D‐printed electrode can retain an areal capacity of 3.1 mAh cm^−2^ with a high cycling stability after 150 cycles at 1.0 C. More impressively, the 3D‐printed Co/Co—N@NPCF‐S electrode possessing an elevated sulfur loading of 7.1 mg cm^−2^ can still stably operate over 80 cycles at 0.2 C, realizing a high areal capacity of 6.4 mAh cm^−2^ (Figure 6h). The excellent electrochemical performance of Co/Co—N@NPCF‐S electrodes even in the case of high sulfur loadings and low electrolyte/sulfur ratios is not only due to the enhanced electron/ion transport kinetics caused by the 3D printed hierarchically porous and conductive framework. The embedded Co nanoparticles and Co—N—C heterointerface within the Co/Co—N@NPCF hybrid also make outstanding contributions to inhibiting LiPSs shuttle behavior and accelerating conversion dynamics.

## 3 Conclusion

In summary, we have demonstrated a general strategy for fabricating a strongly coupled hybrid with continuous metal–nitrogen–carbon heterointerface by bonding metal nanoparticles (Co, Fe, and Ni) with the nitrogen‐doped carbon nanofiber matrix to serve as the sulfur host material. Theoretical calculations accompanied by experimental results certify the pivotal role of interfacial Co—N—C bridging bonds in regulating electron redistribution and inducing charge transfer between the heterogeneous phase, and thus promoting the chemical adsorption toward LiPSs. Besides, the mesoporous carbon nanofiber and embedded Co nanoparticles with electrocatalytic activity are unveiled to stabilize the electrode structure, boost sulfur electrochemistry and achieve high sulfur utilization. As a result, the Co/Co—N@NPCF‐S electrode harvests a superb rate performance (781 mAh g^−1^ at 2.0 C) and prolonged cycling lifespan (capacity decay rate of 0.032% per cycle over 300 cycles). More encouragingly, the Co/Co—N@NPCF‐S electrode with a high sulfur loading of 7.1 mg cm^−2^ based on the 3D printing technique can realize a high areal capacity of 6.4 mAh cm^−2^ at 0.2 C under the lean‐electrolyte condition. We expect this work may provide new insights for the construction of advanced strongly coupled host materials for achieving high‐performance Li—S batteries.

## Conflict of Interest

The authors declare no conflict of interest.

## Supporting information

Supporting Information

## Data Availability

Research data are not shared.

## References

[1] G. Li , Z. Chen , J. Lu , Chem 2018, 4, 3.

[2] S. H. Chung , A. Manthiram , Adv. Mater. 2019, 31, 1901125. 10.1002/adma.201901125 31081272

[3] A. Manthiram , Y. Fu , S. H. Chung , C. Zu , Y. S. Su , Chem. Rev. 2014, 114, 11751. 25026475 10.1021/cr500062v

[4] L. Zhou , D. L. Danilov , R. A. Eichel , P. H. L. Notten , Adv. Energy Mater. 2020, 11, 2001304.

[5] Q. Pang , X. Liang , C. Y. Kwok , L. F. Nazar , Nat. Energy 2016, 1, 16132.

[6] Y. Yao , H. Wang , H. Yang , S. Zeng , R. Xu , F. Liu , P. Shi , Y. Feng , K. Wang , W. Yang , X. Wu , W. Luo , Y. Yu , Adv. Mater. 2020, 32, 1905658. 10.1002/adma.201905658 31830338

[7] W. Yao , W. Zheng , J. Xu , C. Tian , K. Han , W. Sun , S. Xiao , ACS Nano 2021, 15, 7114. 33764730 10.1021/acsnano.1c00270

[8] H. Wei , J. Liu , Y. Liu , L. Wang , L. Li , F. Wang , X. Ren , F. Ren , Compos. Commun. 2021, 28, 100973.

[9] W. P. Wang , J. Zhang , J. Chou , Y. X. Yin , Y. You , S. Xin , Y. G. Guo , Adv. Energy Mater. 2021, 11, 2000791.

[10] J. Lei , T. Liu , J. Chen , M. Zheng , Q. Zhang , B. Mao , Q. Dong , Chem 2020, 6, 2533.

[11] M. Y. Qi , Y. S. Xu , S. J. Guo , S. D. Zhang , J. Y. Li , Y. G. Sun , K. C. Jiang , A. M. Cao , L. J. Wan , Small Sci. 2021, 1, 2100066. 10.1002/smsc.202100066 PMC11935911 40212956

[12] W. Li , K. Chen , Q. Xu , X. Li , Q. Zhang , J. Weng , J. Xu , Angew. Chem., Int. Ed. 2021, 133, 21682.

[13] J. He , A. Manthiram , Adv. Energy Mater. 2020, 10, 2002654. 10.1002/aenm.202001972 PMC8216142 34158810

[14] C. X. Zhao , X. Y. Li , M. Zhao , Z. X. Chen , Y. W. Song , W. J. Chen , J. N. Liu , B. Wang , X. Q. Zhang , C. M. Chen , B. Q. Li , J. Q. Huang , Q. Zhang , J. Am. Chem. Soc. 2021, 143, 19865. 34761937 10.1021/jacs.1c09107

[15] L. Du , H. Wang , M. Yang , L. Liu , Z. Niu , Small Struct. 2020, 1, 2000047.

[16] J. Q. Huang , X. F. Liu , Q. Zhang , C. M. Chen , M. Q. Zhao , S. M. Zhang , W. Zhu , W. Z. Qian , F. Wei , Nano Energy 2013, 2, 314.

[17] J. Li , Y. Qu , C. Chen , X. Zhang , M. Shao , Nanoscale 2021, 13, 15. 33325951 10.1039/d0nr06732f

[18] H. Li , W. Xue , W. Xu , L. Wang , T. Liu , Compos. Commun. 2021, 24, 100675.

[19] M. Yu , S. Zhou , Z. Wang , Y. Wang , N. Zhang , S. Wang , J. Zhao , J. Qiu , Energy Storage Mater. 2019, 20, 98.

[20] Z. P. Wu , X. F. Lu , S. Q. Zang , X. W. Lou , Adv. Funct. Mater. 2020, 30, 1910274.

[21] Q. Shi , S. Hwang , H. Yang , F. Ismail , D. Su , D. Higgins , G. Wu , Mater. Today 2020, 37, 93.

[22] T. Zhang , W. Zong , Y. Ouyang , Y. Wu , Y. E. Miao , T. Liu , Adv. Fiber Mater. 2021, 3, 229.

[23] G. Zhou , S. Zhao , T. Wang , S. Z. Yang , B. Johannessen , H. Chen , C. Liu , Y. Ye , Y. Wu , Y. Peng , Nano Lett. 2019, 20, 1252. 10.1021/acs.nanolett.9b04719 31887051

[24] L. Su , J. Zhang , Y. Chen , W. Yang , J. Wang , Z. Ma , G. Shao , G. Wang , Nano Energy 2021, 85, 105981.

[25] T. Chen , B. Cheng , G. Zhu , R. Chen , Y. Hu , L. Ma , H. Lv , Y. Wang , J. Liang , Z. Tie , Nano Lett. 2017, 17, 437. 28073275 10.1021/acs.nanolett.6b04433

[26] S. Liu , J. Li , X. Yan , Q. Su , Y. Lu , J. Qiu , Z. Wang , X. Lin , J. Huang , R. Liu , B. Zheng , L. Chen , R. Fu , D. Wu , Adv. Mater. 2018, 30, 1706895. 10.1002/adma.201706895 29423940

[27] C. Yan , Y. Zhu , Z. Fang , C. Lv , X. Zhou , G. Chen , G. Yu , Adv. Energy Mater. 2018, 8, 1800762.

[28] C. Zhu , T. Liu , F. Qian , W. Chen , S. Chandrasekaran , B. Yao , Y. Song , E. B. Duoss , J. D. Kuntz , C. M. Spadaccini , M. A. Worsley , Y. Li , Nano Today 2017, 15, 107.

[29] N. Wang , X. Zhang , Z. Ju , X. Yu , Y. Wang , Y. Du , Z. Bai , S. Dou , G. Yu , Nat. Commun. 2021, 12, 4519. 34312377 10.1038/s41467-021-24873-4 PMC8313709

[30] Y. Hu , W. Chen , T. Lei , Y. Jiao , J. Huang , A. Hu , C. Gong , C. Yan , X. Wang , J. Xiong , Adv. Energy Mater. 2020, 10, 2000082.

[31] S. Zhou , I. Usman , Y. Wang , A. Pan , Energy Storage Mater. 2021, 38, 141.

[32] Y. Pang , Y. Cao , Y. Chu , M. Liu , K. Snyder , D. MacKenzie , C. Cao , Adv. Funct. Mater. 2020, 30, 1906244.

[33] J. Wang , Q. Sun , X. Gao , C. Wang , W. Li , F. B. Holness , M. Zheng , R. Li , A. D. Price , X. Sun , T. K. Sham , X. Sun , ACS Appl. Mater. Interfaces 2018, 10, 39794. 30372018 10.1021/acsami.8b14797

[34] C. Wei , M. Tian , M. Wang , Z. Shi , L. Yu , S. Li , Z. Fan , R. Yang , J. Sun , ACS Nano 2020, 14, 16073. 33156985 10.1021/acsnano.0c07999

[35] K. Shen , H. Mei , B. Li , J. Ding , S. Yang , Adv. Energy Mater. 2018, 8, 1701527.

[36] X. Gao , Q. Sun , X. Yang , J. Liang , A. Koo , W. Li , J. Liang , J. Wang , R. Li , F. B. Holness , A. D. Price , S. Yang , T. K. Sham , X. Sun , Nano Energy 2019, 56, 595.

[37] J. Shen , X. Xu , J. Liu , Z. Wang , S. Zuo , Z. Liu , D. Zhang , J. Liu , M. Zhu , Adv. Energy Mater. 2021, 11, 2100673.

[38] Y. Tong , P. Chen , T. Zhou , K. Xu , W. Chu , C. Wu , Y. Xie , Angew. Chem., Int. Ed. 2017, 56, 7121. 10.1002/anie.201702430 28523861

[39] S. H. Chung , L. Luo , A. Manthiram , ACS Energy Lett. 2018, 3, 568.

[40] Y. W. Song , Y. Q. Peng , M. Zhao , Y. Lu , J. N. Liu , B. Q. Li , Q. Zhang , Small Sci. 2021, 1, 2100042. 10.1002/smsc.202100042 PMC11935945 40212959

[41] J. J. Chen , R. M. Yuan , J. M. Feng , Q. Zhang , J. X. Huang , G. Fu , M. S. Zheng , B. Ren , Q. F. Dong , Chem. Mater. 2015, 27, 2048.

[42] S. Deng , Q. Zhang , Q. Huang , D. Tang , P. Mei , Y. Yang , Compos. Commun. 2022, 29, 101019.

[43] L. Wang , T. Liu , T. Wu , J. Lu , Exploration 2021, 1, 20210130. 10.1002/EXP.20210130 PMC10190967 37323695

[44] K. Fu , Y. Wang , C. Yan , Y. Yao , Y. Chen , J. Dai , S. Lacey , Y. Wang , J. Wan , T. Li , Adv. Mater. 2016, 28, 2587. 26833897 10.1002/adma.201505391

