# Supplementary material for: A Universal Spinning‐Coordinating Strategy to Construct Continuous Metal–Nitrogen–Carbon Heterointerface with Boosted Lithium Polysulfides Immobilization for 3D‐Printed Li—S Batteries
Source: Adv Sci (Weinh). 2022 Jul 21;9(26):2203181. doi: 10.1002/advs.202203181 (PMC9475505; doi:10.1002/advs.202203181)
Supplement: Supplementary file 1 — Supporting Information [file ADVS-9-2203181-s001.pdf]

ADVANCED SCIENCE
Open Access

Supporting Information

for *Adv. Sci.*, DOI 10.1002/advs.202203181

A Universal Spinning-Coordinating Strategy to Construct Continuous Metal–Nitrogen–Carbon Heterointerface with Boosted Lithium Polysulfides Immobilization for 3D-Printed Li–S Batteries

Yue Ouyang, Wei Zong, Xiaobo Zhu, Lulu Mo, Guojie Chao, Wei Fan, Feili Lai, Yue-E Miao, Tianxi Liu and Yan Yu**

A Universal Spinning-Coordinating Strategy to Construct Continuous Metal-Nitrogen-Carbon Heterointerface with Boosted Lithium Polysulfides Immobilization for 3D-Printed Li-S Batteries

Yue Ouyang, Wei Zong, Xiaobo Zhu, Lulu Mo, Guojie Chao, Wei Fan, Feili Lai, Yue-E Miao, Tianxi Liu, and Yan Yu**

Y. Ouyang, W. Zong, X. B. Zhu, L. L. Mo, Prof. W. Fan, Prof. Y. E. Miao, Prof. T. X. Liu
State Key Laboratory for Modification of Chemical Fibers and Polymer Materials, College of Materials Science and Engineering, Donghua University, 2999 North Renmin Road, Shanghai 201620, P. R. China.
E-mail: yuee_miao@dhu.edu.cn

Prof. Y. Yu
Hefei National Research Center for Physical Sciences at the Microscale, Department of Materials Science and Engineering, National Synchrotron Radiation Laboratory, CAS Key Laboratory of Materials for Energy Conversion, University of Science and Technology of China, Hefei, Anhui 230026, P. R. China.
E-mail: yanyumse@ustc.edu.cn

Dr. G. J. Chao, Prof. T. X. Liu
The Key Laboratory of Synthetic and Biological Colloids, Ministry of Education, School of Chemical and Material Engineering, Jiangnan University, Wuxi Jiangsu 214122, P. R. China.

Dr. F. L. Lai
Department of Chemistry, KU Leuven, Celestijnenlaan 200F, Leuven 3001, Belgium.

Experimental section

Materials

Polystyrene (PS, $M_w \approx 260{,}000$) was purchased from J&K Chemical Ltd. Dopamine hydrochloride (98%) and tris(hydroxymethyl) aminomethane hydrochloride (TRIS·HCl, 99%) were obtained from Aladdin Chemical Reagent. Iron(II) acetate was provided by Tokyo Chemical Industry. Cobalt acetate tetrahydrate ($Co(Ac)_2 \cdot 4H_2O$, 99%), nickel acetate tetrahydrate ($Ni(Ac)_2 \cdot 4H_2O$, 99%), sublimed sulfur, and *N*,*N*-dimethylformamide (DMF) were supplied by Sinopharm Chemical Reagent Co., Ltd. All the reagents were of analytical grade and used without further purification.

Preparation of M/M-N@NPCF-S composite

First, PS was dissolved in DMF with vigorous stirring at room temperature for 12 h to prepare a 30 wt% homogeneous solution for electrospinning. Then, the PS solution was placed in a syringe connected with a 0.5 mm metal needle and directly electrospun into the liquid collector filled with ethanol to obtain the porous PS fibers. To be specific, a voltage of 11 kV was applied to the spinneret, while the distance between the needle and collector was set as 10 cm. Benefitting from the high affinity and excellent adhesion property of dopamine to various substrates, the as-collected PS fibers were then transferred to an aqueous solution of dopamine (2 mg mL^{-1} in 10 mM Tris buffer, pH = 8.5) and metal salt ($Co(Ac)_2 \cdot 4H_2O$, $Ni(Ac)_2 \cdot 4H_2O$, and Iron(II) acetate, 0.1 M), and mildly stirred for 24 h, to acquire PS fibers in-situ coated with polydopamine-metal^{n+} (PS@PDA-Metal^{n+}). Then, PS@PDA-Metal^{n+} was calcinated in high-purity nitrogen (N_2) environment at 700 °C for 2 h with a heating rate of 5 °C

min^{-1} to make the PS template completely decomposed. The coordinated PS@PDA-Metal^{n+} organic/inorganic precursor was simultaneously converted to a strongly coupled hybrid material comprised of nitrogen-doped porous carbon nanofiber embedded with cobalt nanoparticles, which was signed as M/M-N@NPCF. In addition, an aqueous solution of dopamine (2 mg mL^{-1} in 10 mM Tris buffer, pH = 8.5) and $Co(Ac)_2 \cdot 4H_2O$ (0.1 M) without the addition of porous PS fiber template was prepared and mildly stirred for 24 h to collect PDA@Co^{2+} powder. Then, the PDA@Co^{2+} powder was carbonized at the same condition to obtain a comparison sample of nitrogen-doped carbon embedded with cobalt nanoparticles (Co/Co-N@NC).

Based on a melt-diffusion method, the mixed powder, consisting of the host material of M/M-N@NPCF hybrid and sulfur at a weight ratio of 1:2, was transferred to a sealed vessel after grinding for several minutes, and heated at 155 °C for 12 h to fabricate the M/M-N@NPCF-S composite. For comparison, the sulfur loaded nitrogen-doped porous carbon fiber (NPCF-S) was prepared by the same method except for immersing metal salt solution.

Preparation of plain M/M-N@NPCF-S cathode

Typically, the electrode materials of M/M-N@NPCF-S and NPCF-S were respectively mixed with carbon black and poly(vinyl difluoride) (PVDF) at a weight ratio of 8:1:1. The slurry was then cast onto aluminum foils and vacuum dried at 50 °C for 12 h to obtain the plain cathodes. The CB-S electrode was also prepared by mixing sulfur, Super P and PVDF with a mass ratio of 6:3:1. The sulfur loading in the

electrodes was basically fixed at 1.1 mg cm^{-2} without specific illustration.

Preparation of 3D-printed Co/Co-N@NPCF-S cathode

Firstly, 50 mg of graphene oxide (GO) powder was sonicated in 2 mL deionized water for 2 h to obtain GO solution. Afterwards, 450 mg of Co/Co-N@NPCF-S and several drops of water were added into the above solution and stirred for 12 h to form the uniform ink. The as-prepared ink was then transferred into a syringe tube attached with a blunt-tip needle (inner diameters of needle tips: 0.46 mm, 26 Ga) for direct ink writing 3D printing. And the 3D printing process was conducted by a modified 3D printing system (RZC-30WK, Dongguan origin Automation Technology Co. Ltd). Under the appropriate printing speed and air pressure, the ink was printed as electrodes of tunable thicknesses with an area of 1.0 cm^{-2}. Finally, the 3D-printed architectures were freeze-dried to obtain the 3D-printed Co/Co-N@NPCF-S cathodes with various sulfur loadings.

Characterizations

Morphology of the samples was observed with the field-emission scanning electron microscope (FESEM, JSM 7500F) and field-emission transmission electron microscope (FETEM, Talos F200S). X-ray diffraction (XRD) patterns were obtained on an X'Pert PRO X-ray diffractometer at a current of 40 mA and voltage of 40 kV with Cu K_{α} radiation. X-ray photoelectron spectroscopy (XPS) was examined by the Thermo Scientific ESCALAB 250Xi equipped with an Al K_{α} X-ray source at an energy of 1486.6 eV. Brunauer-Emmett-Teller (BET) nitrogen adsorption/desorption isotherms were measured by using a Quantachrome Autosorb-iQ/MP®XR system.

Thermogravimetric analysis (TGA) was carried out on a NETZSCH TG209F1 Libra device.

For the visualized adsorption test, Li_2S_6 as a representative of LiPSs, was prepared by mixing lithium sulfides (Li_2S) and sublimed sulfur at a molar ratio of 1:5 in *1,3*-dioxolane/*1,2*-dimethoxyethane (DOL/DME) (1:1, v/v) followed by vigorous stirring for 24 h. A 5 mM Li_2S_6 solution was used for the adsorption test. Typically, Co/Co-N@NPCF and NPCF were added to 3 mL of Li_2S_6 solutions respectively, with the pure Li_2S_6 solution as a comparison. Then, digital photos of the macroscopic adsorption toward LiPSs by different materials were taken after resting for 12 h.

Electrochemical measurements

The coin cells were assembled in an argon-filled glove box with lithium foil as the counter electrode, 1.0 M lithium bis(trifluoromethanesulfonyl)imide (LiTFSI) in DOL/DME (1:1, v/v) containing 2% $LiNO_3$ as the electrolyte, a Celgard 2500 membrane as separator, and a plain or 3D-printed cathode as the working electrode. The galvanostatic discharge/charge tests were performed between 1.7 - 2.8 V (vs. Li/Li^+) by a LAND 2001A battery testing system. Cyclic voltammetry (CV) curves were carried out on an ARBIN BT2000 system in the voltage range of 1.7 - 2.8 V at different scan rates. Electrochemical impedance spectra (EIS) measurements were carried out by an electrochemical workstation in the frequency range from 10^5 Hz to 1 Hz under the automatic sweep mode.

The electrodes used for symmetrical batteries were prepared without elemental sulfur. The host material of Co/Co-N@NPCF or NPCF was dispersed in NMP with

PVDF binder at a mass ratio of 9:1 to form a uniform slurry. The slurry was coated on the aluminum foils and dried at 80 °C under vacuum for 12 h to prepare the electrode disks as both working and counter electrodes. In addition, a Celgard 2500 membrane was used as the separator and 40 μL of 0.5 M Li_2S_6 in DOL/DME (1:1, v/v) solution with 1.0 M LiTFSI was added as electrolyte. CV measurements of the symmetrical batteries were performed at a scan rate of 50 mV s^{-1} between -0.8 V and 0.8 V.

The electrodes used for Li_2S nucleation measurements were similarly prepared as above. A Li_2S_8 solution was prepared by mixing Li_2S and sulfur at a mass ratio of 1:7 in tetraglyme solution with 1 M LiTFSI. Co/Co-N@NPCF or NPCF cathodes was used as the working electrode and the bare lithium foil works as the counter electrode. 20 μL of Li_2S_8 solution was dropped onto the working electrodes, while 20 μL of 1.0 M LiTFSI (DOL/DME, 1:1, v/v) electrolyte was dropped onto the Li anode. The cell assembly was performed in an Ar-filled glovebox with a Celgard 2500 membrane as the separator. The cells were galvanostatically discharged to 2.06 V at 0.112 mA and then kept potentiostatically at 2.05 V for Li_2S nucleation and growth until the current dropped below 10^{-5} A.

The in-situ reaction cell with a quartz window on negative case was used for in-situ Raman spectroscopy analysis. A hole was punched on Celgard separator and Li metal anode to allow the laser to directly shine on the Co/Co-N@NPCF-S cathode and separator facing the Li anode side (Figure S19, Supporting Information). The discharge/charge rate was set as 0.15 C and Raman signals were recorded by a

Ramantouch microspectrometer (Nanophoton Inc., Osaka, Japan) with a 532 nm excitation laser.

Computational method

The Vienna Ab initio Simulation Package (VASP) was employed to perform all density functional theory (DFT) calculations within the generalized gradient approximation (GGA) using the Perdew-Burke-Ernzerhof (PBE) functional. The projected augmented wave (PAW) potentials were chosen to describe the ionic cores and take valence electrons into account using a plane wave basis set with a kinetic energy cut-off of 400 eV. Geometry optimizations were performed with the force convergency smaller than 0.05 eV/Å. Monkhorst-Pack k-points of 2×2×1 were applied for all the calculations. All the atoms are relaxed in all the calculations and the corresponding lattice parameter: a = b = 14.89797 Å, c = 20 Å, α = β = 90°, γ = 120°. The binding energy (ΔE) of Li_2S_n on the surface was defined as:

$$\Delta E = E(Li_2S_n + sub) - E(sub) - E(Li_2S_n) \quad (1)$$

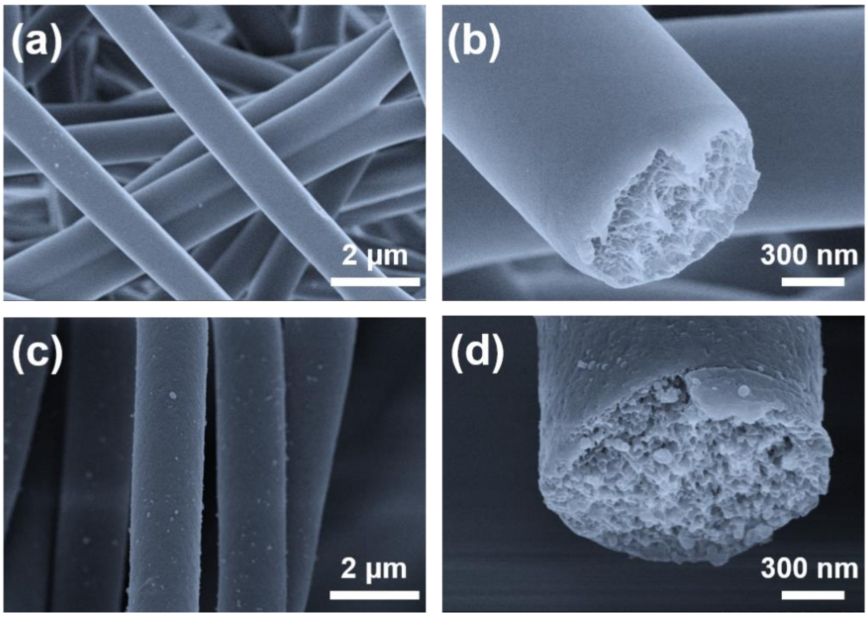

Figure S1. SEM images of (a-b) PS and (c-d) PS@PDA-metal^{n+} fibers.

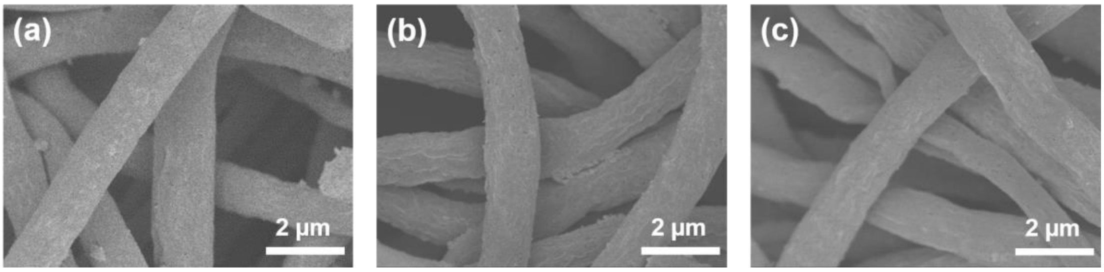

Figure S2. SEM images of (a) Co/Co-N@NPCF, (b) Fe/Fe-N@NPCF and (c) Ni/Ni-N@NPCF hybrids.

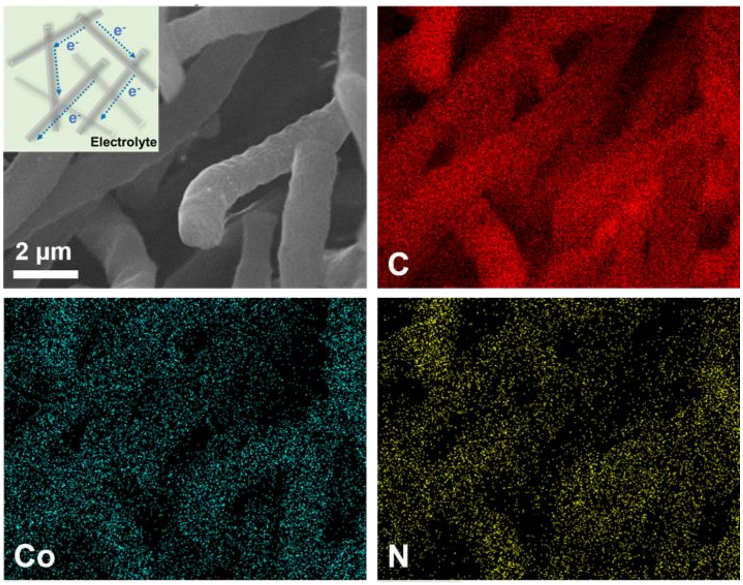

Figure S3. SEM and corresponding EDX elemental mappings of Co/Co-N@NPCF hybrid.

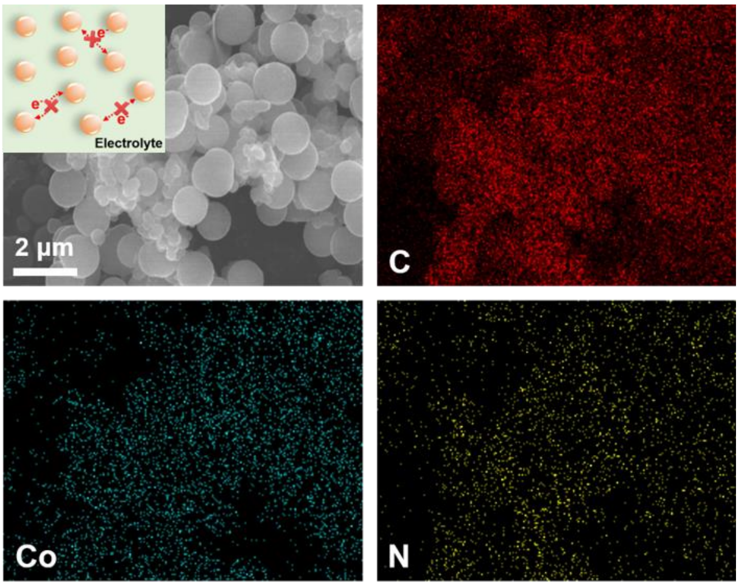

Figure S4. SEM and corresponding EDX elemental mappings of Co/Co-N@NC composite.

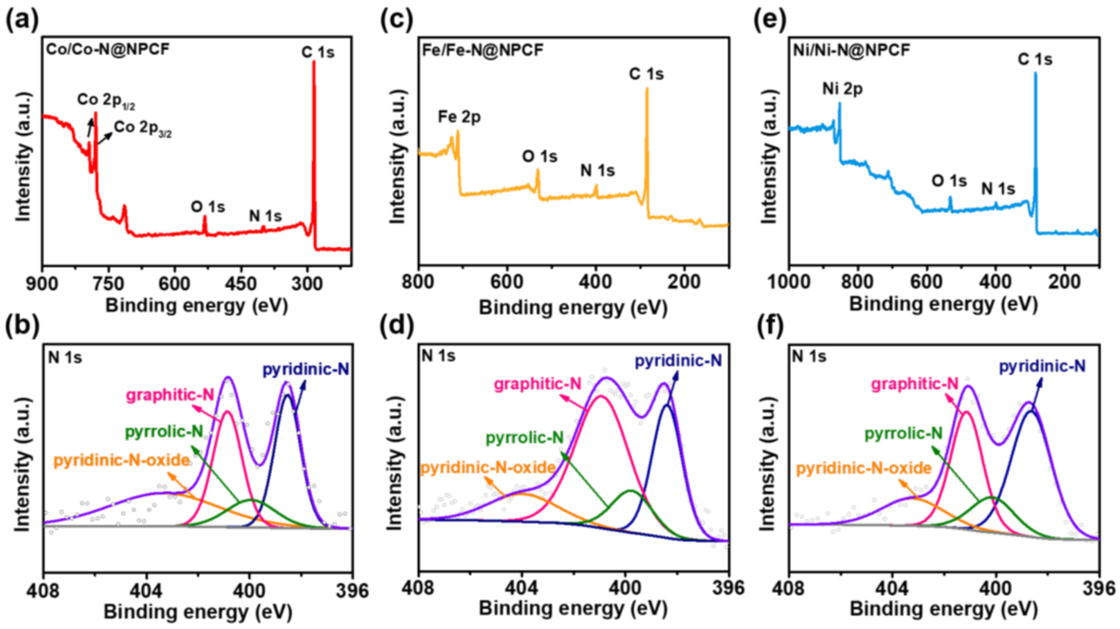

Figure S5. XPS spectra of (a-b) Co/Co-N@NPCF, (c-d) Fe/Fe-N@NPCF and (e-f) Ni/Ni-N@NPCF hybrids, respectively.

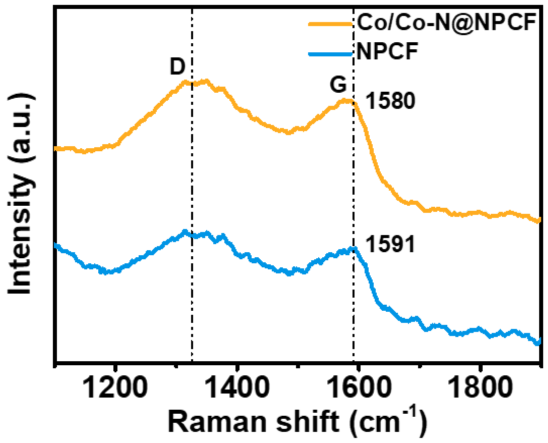

Figure S6. Raman spectra of Co/Co-N@NPCF and NPCF, respectively.

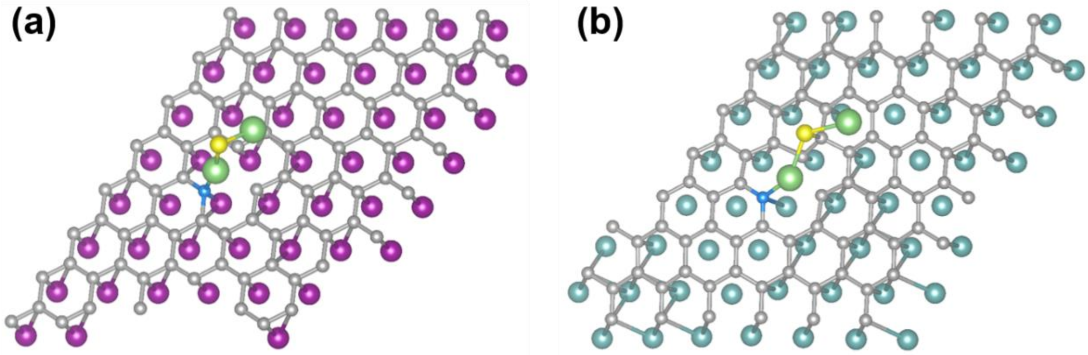

Figure S7. Adsorption configuration of (a) Li_2S - Fe/Fe-N@NPCF and (b) Li_2S - Ni/Ni-N@NPCF.

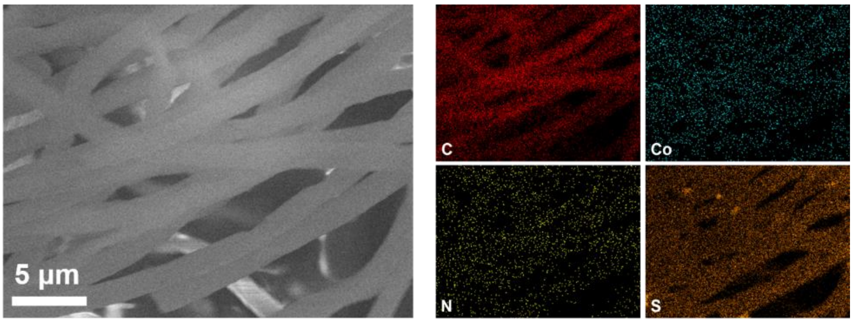

Figure S8. SEM and corresponding EDX elemental mappings of Co/Co-N@NPCF-S composite.

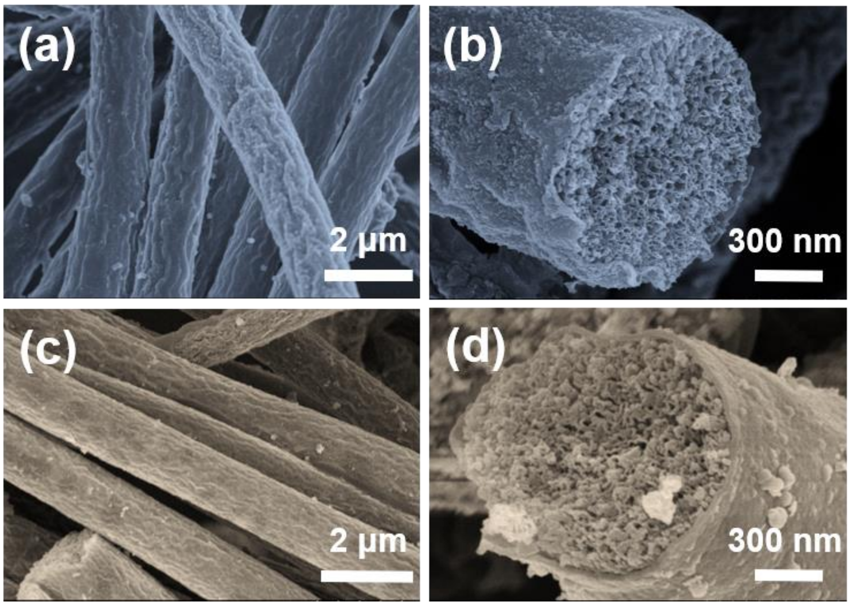

Figure S9. SEM images of (a-b) NPCF and (c-d) NPCF-S composites.

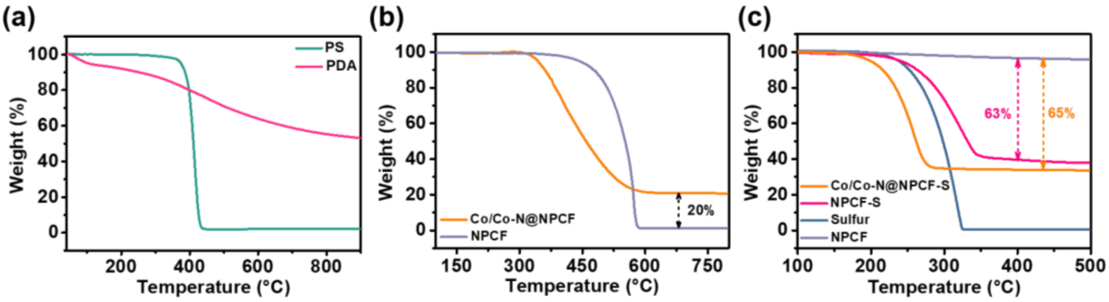

Figure S10. TGA curves of (a) PS and PDA, and (b) NPCF and Co/Co-N@NPCF measured in air atmosphere; and (c) sulfur, NPCF, NPCF-S and Co/Co-N@NPCF-S composites measured in N_2 atmosphere.

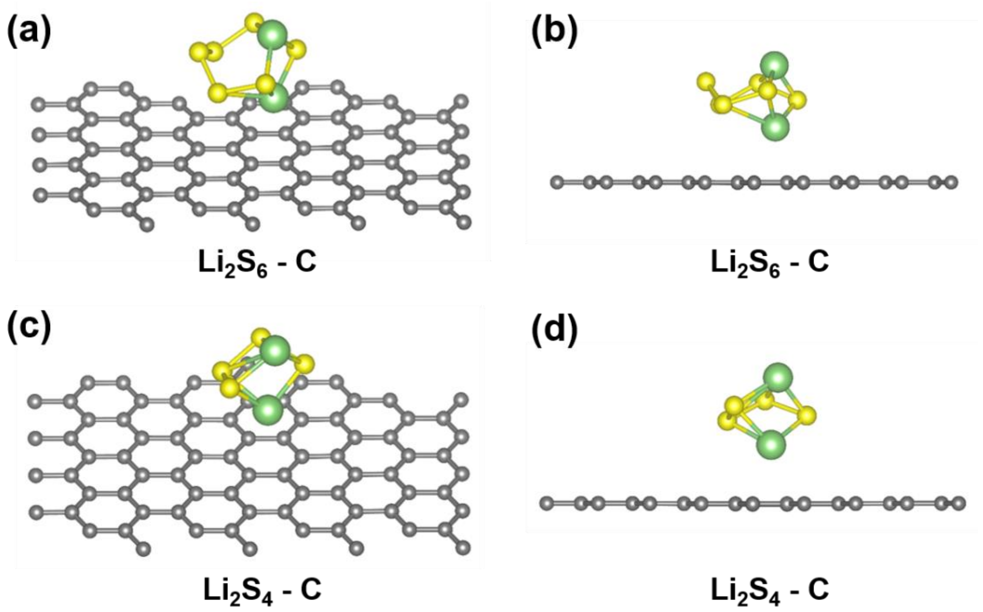

Figure S11. Adsorption configurations of: (a-b) Li_2S_6 - C, (c-d) Li_2S_4 - C. The Li, C, and S atoms are denoted by green, grey, and yellow balls, respectively.

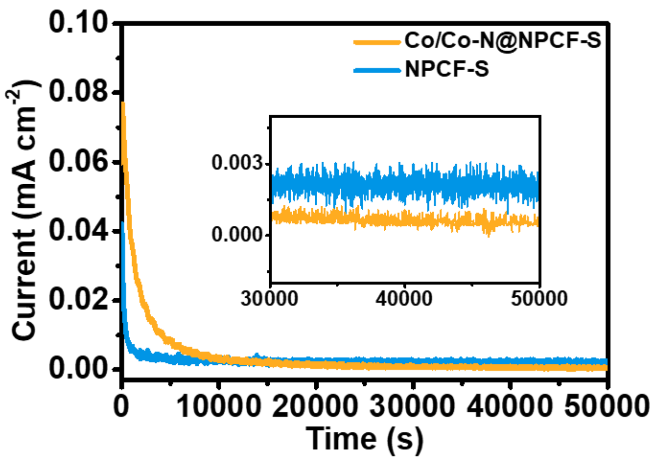

Figure S12. Shuttle current of Co/Co-N@NPCF-S and NPCF-S electrodes.

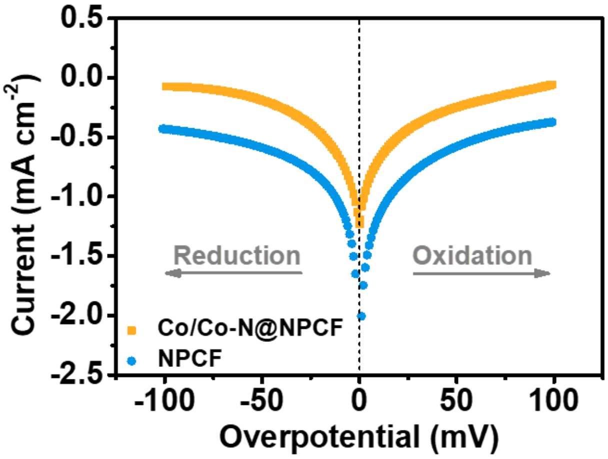

Figure S13. Tafel plots of Co/Co-N@NPCF and NPCF-based cells.

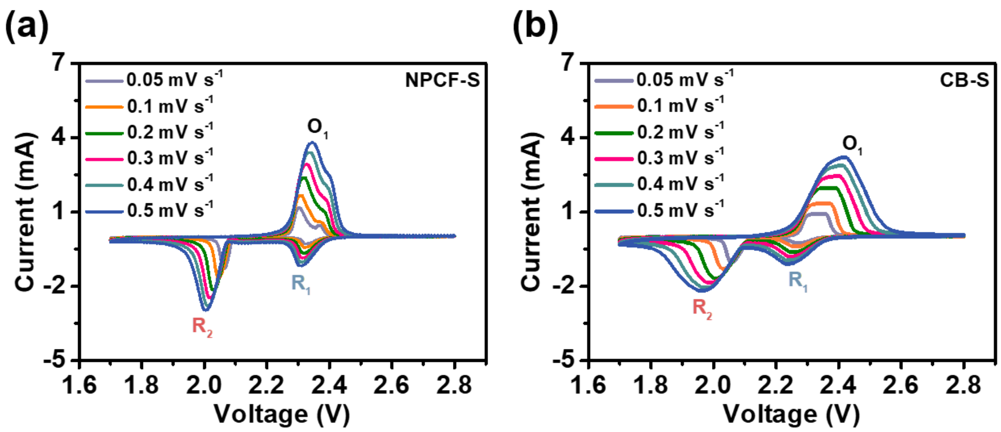

Figure S14. CV curves of (a) NPCF-S and (b) CB-S electrodes under various scan rates ranging from 0.05 to 0.5 mV s^{-1} between 1.7 and 2.8 V.

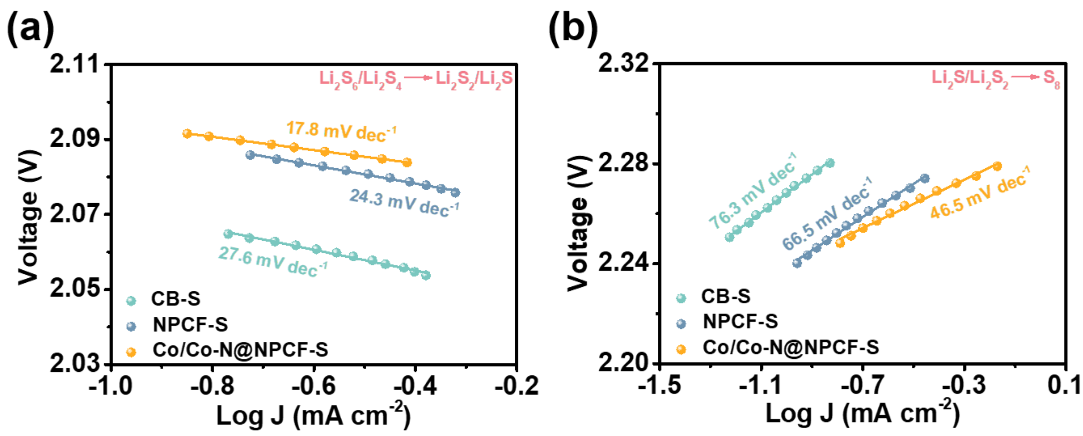

Figure S15. Tafel plots for the conversion reactions with Co/Co-N@NPCF-S, NPCF-S and CB-S electrodes.

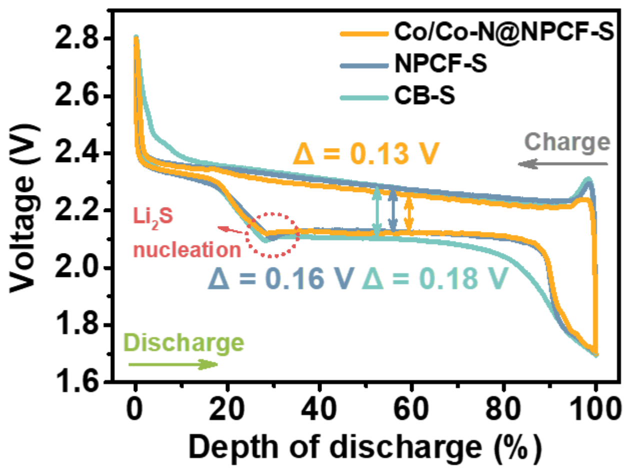

Figure S16. In-depth charge/discharge curves of Co/Co-N@NPCF-S, NPCF-S and CB-S electrodes.

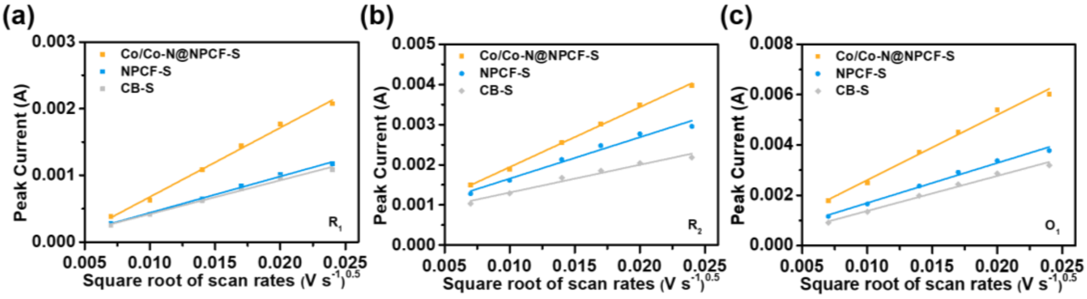

Figure S17. Curves of the CV peak current for (a) the first cathodic reduction reaction (R_1, S_8-Li_2S_4), (b) the second cathodic reduction reaction (R_2, Li_2S_4-Li_2S), and (c) the anodic oxidation reaction (O_1) versus the square root of scan rates.

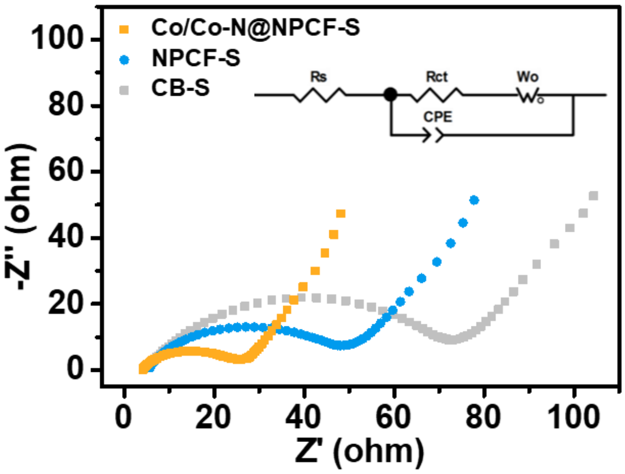

Figure S18. EIS spectra of Co/Co-N@NPCF-S, NPCF-S and CB-S electrodes, respectively.

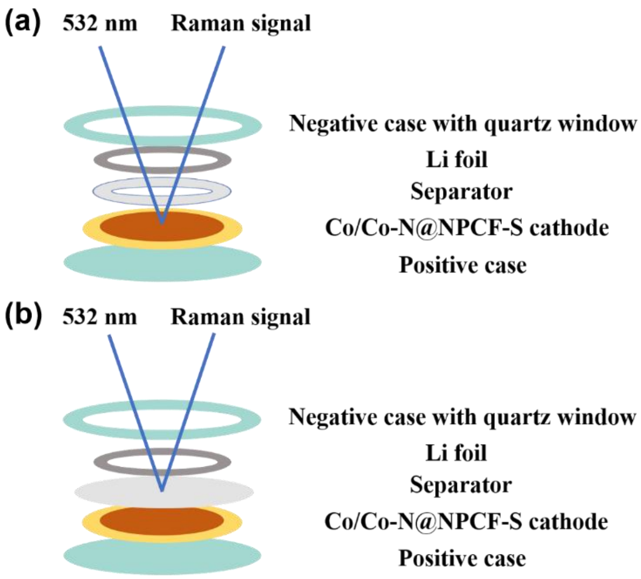

Figure S19. Schematic illustration of the reaction cells toward in-situ Raman tests.

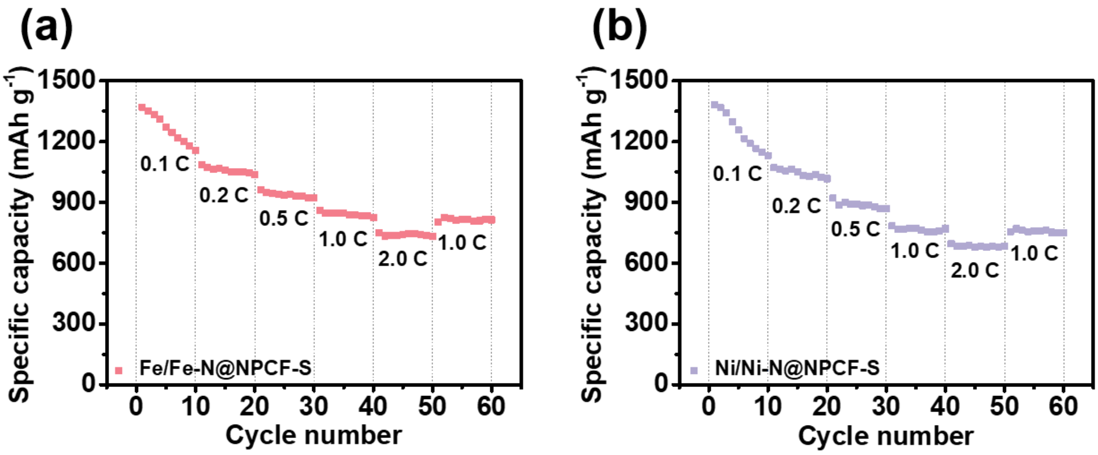

Figure S20. Rate performance of (a) Fe/Fe-N@NPCF-S and (b) Ni/Ni-N@NPCF-S electrodes, respectively.

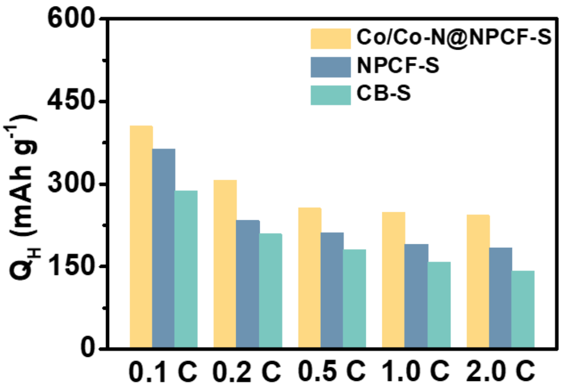

Figure S21. Comparison of the Q_H capacities of Co/Co-N@NPCF-S, NPCF-S and CB-S electrodes.

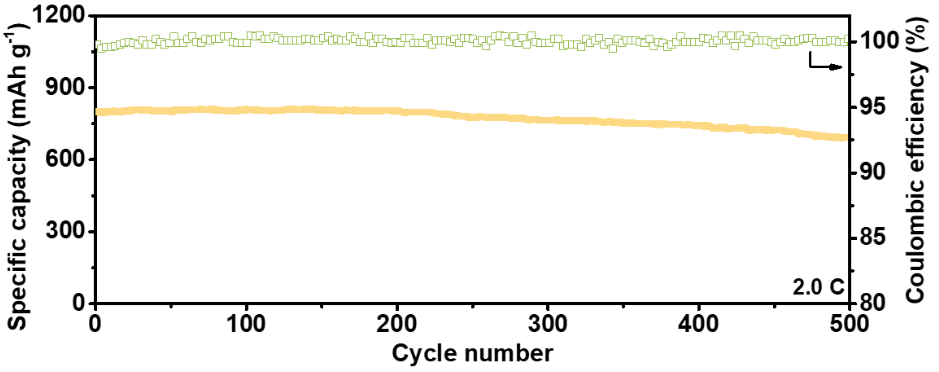

Figure S22. Cycling performance of Co/Co-N@NPCF-S electrode at 2.0 C.

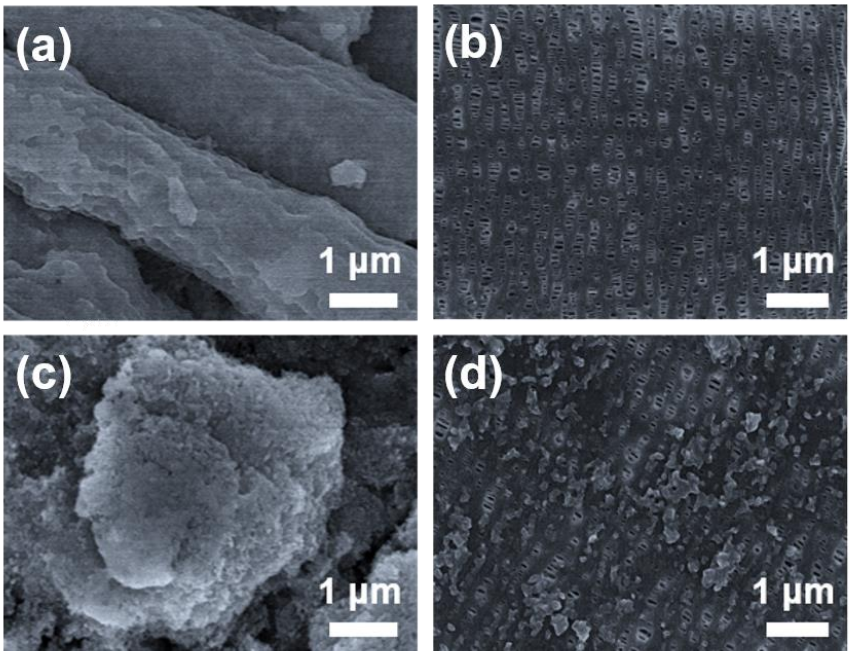

Figure S23. SEM images of the electrode surface and the corresponding separator in (a-b) Co/Co-N@NPCF-S based battery and (c-d) CB-S based battery after cycling process.

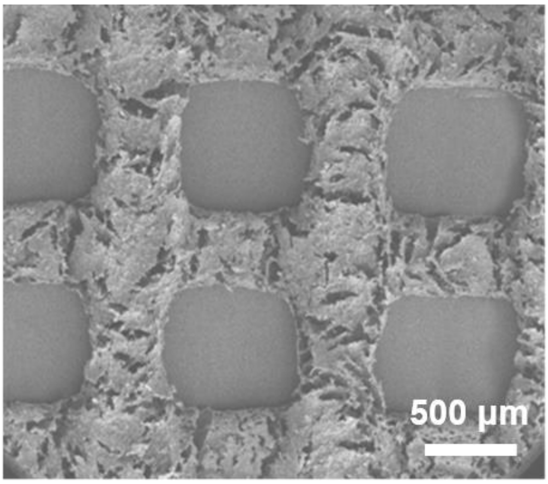

Figure S24. SEM image of 3D-printed Co/Co-N@NPCF-S electrode.

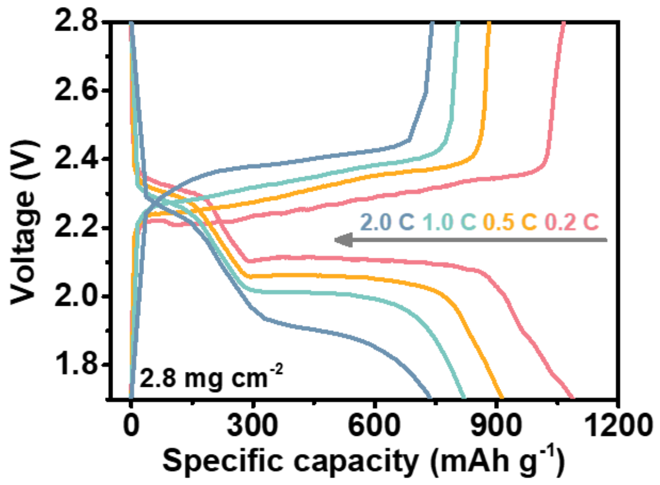

Figure S25. Voltage profiles of 3D-printed Co/Co-N@NPCF-S electrode at various current densities.

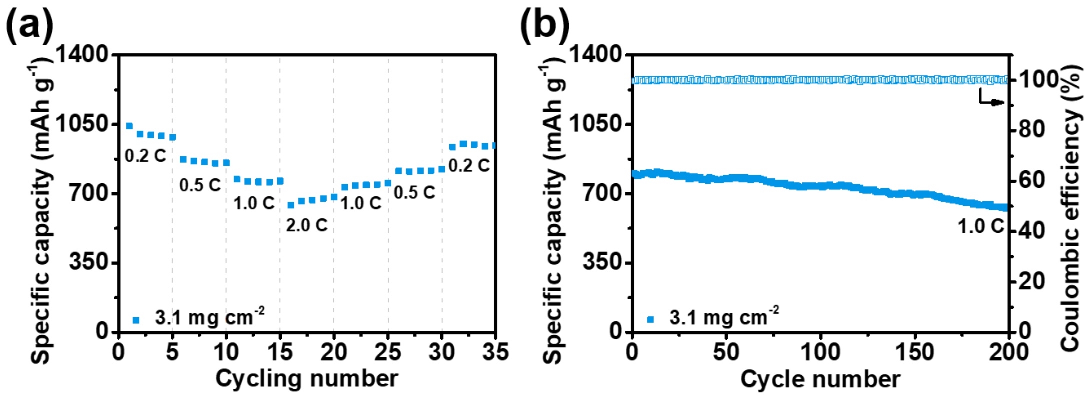

Figure S26. (a) Rate capability and (b) cycling performance of the plain Co/Co-N@NPCF-S electrode.

Table S1. Simulation results of the kinetic parameters of Co/Co-N@NPCF-S, NPCF-S and CB-S electrodes.

**Parameters**	**R_s (Ω)**	**R_{ct} (Ω)**
**Co/Co-N@NPCF-S**	4.3	20.9
**NPCF-S**	5.8	41.0
**CB-S**	6.2	65.2

As shown in Figure S18, all the Nyquist plots of the batteries were qualitatively analyzed by Z-view software. The intercept on the real axis at the high frequency section represents the ohmic resistance (R_s), while the semicircle at the high to medium frequency region is ascribed to the charge transfer resistance (R_{ct}).

Table S2. Comparisons of the electrochemical performance of the as-developed 3D-printed Co/Co-N@NPCF-S based battery with recently reported 3D-printed Li-S batteries.

Cathode	Sulfur loading (mg cm^{-2})	Initial areal capacity (mAh cm^{-2})	Capacity retention (%)	Refs
3DP *N*-pTi$_3$C$_2$T$_x$/S	5.55	6.06 (0.2 C)	/	[1]
	7.56	(0.2 C)	85 (250)	
	8.86	/	/	
	12.02	/	/	
3DP CoFe-MCS	3.9	3.13 (0.5 C)	88.8 (100)	[2]
	5.2	5.2 (0.1 C)	76.9 (45)	
	7.7	6.0 (0.1 C)	83.3 (30)	
BP-2000	3.0	3.6 (0.2 C)	63.5 (200)	[3]
	5.5	5.0 (2.0 C)	61.7 (200)	
3DP rGO/DIB	2.0	1.63 (0.03 C)	43.3 (50)	[4]
Super P/LaB$_6$	3.6	(1.0 C)	78.3 (350)	[5]
	9.3	7.93 (0.05 C)	88.5 (50)	
3D printed Co/Co-N@NPCF-S	4.5	3.5 (1.0 C)	85.7 (150)	This work
	7.1	6.4 (0.2 C)	92.2 (80)	

References:

[1] C. Wei, M. Tian, Z. Fan, L. Yu, Y. Song, X. Yang, Z. Shi, M. Wang, R. Yang, J. Sun, *Energy Storage Mater.* **2021**, *41*, 141.

[2] Z. Shi, Z. Sun, J. Cai, Z. Fan, J. Jin, M. Wang, J. Sun, *Adv. Funct. Mater.* **2020**, *31*, 2006798.

[3] X. Gao, Q. Sun, X. Yang, J. Liang, A. Koo, W. Li, J. Liang, J. Wang, R. Li, F. B. Holness, A. D. Price, S. Yang, T.-K. Sham, X. Sun, *Nano Energy* **2019**, *56*, 595.

[4] K. Shen, H. Mei, B. Li, J. Ding, S. Yang, *Adv. Energy Mater.* **2018**, *8*, 1701527.

[5] J. Cai, Z. Fan, J. Jin, Z. Shi, Z. Liu, *Nano Energy* **2020**, *75*, 104970.
